# Supplementary material for: Conditioning Regimens in Patients with β-Thalassemia Who Underwent Hematopoietic Stem Cell Transplantation: A Scoping Review
Source: J Clin Med. 2022 Feb 9;11(4):907. doi: 10.3390/jcm11040907 (PMC8876955; doi:10.3390/jcm11040907)
Supplement: Supplementary file 1 [file jcm-11-00907-s001.zip › jcm-1566236-supplementary.pdf]

**Supplementary Table S1. Search strategy:**

| Database | Search strategy |
|---|---|
| pubmed | ("thalassaemia"[All Fields] OR "thalassemia"[MeSH Terms] OR "thalassemia"[All Fields] OR "thalassaemias"[All Fields] OR "thalassemias"[All Fields]) AND ("bone marrow transplantation"[MeSH Major Topic] OR (("haematopoietically"[All Fields] OR "hematopoietic system"[MeSH Terms] OR ("hematopoietic"[All Fields] AND "system"[All Fields]) OR "hematopoietic system"[All Fields] OR "haematopoietic"[All Fields] OR "hematopoietic"[All Fields] OR "hematopoietically"[All Fields]) AND "stem cell transplantation"[MeSH Major Topic])) |
| Web Of Science | ALL FIELDS: (thalassemia) AND ALL FIELDS: (((bone marrow transplant) OR (hematopoietic stem cell transplant))) Timespan: All years. Indexes: SCI-EXPANDED, SSCI, A&HCI, CPCI-S, CPCI-SSH, BKCI-S, BKCI-SSH, ESCI, CCR-EXPANDED, IC. |
| Scopus | TITLE-ABS-KEY ( thalassemia ) AND TITLE-ABS-KEY ( ( bone AND marrow AND transplantation ) OR ( hematopoietic AND stem AND cell AND transplant ) ) ) |
